# Comparing strategies for association mapping in samples with related individuals

**DOI:** 10.1186/1471-2156-6-S1-S98

**Published:** 2005-12-30

**Authors:** Catherine Bourgain

**Affiliations:** 1 INSERM U535, Hopital Paul Brousse, Batiment Leriche B.P.1000, 94817 Villejuif Cedex, France

## Abstract

In this paper, different strategies to test for association in samples with related individuals designed for linkage studies are compared. Because no independent controls are available, a family-based association test and case-control tests corrected for the presence of related individuals in which unaffected relatives are used as controls were tested. When unrelated controls are available, additional strategies including selection of a single case per family considering either all families or a subset of linked families, are also considered. Analyses are performed on the simulated dataset, blind to the answers. The case-control test corrected for the presence of related individuals is the most powerful strategy to detect three loci associated with the disease under study. Using a correction factor for the case-control test performed conditional on the marker information rather than unconditional does not impact the power significantly.

## Background

Different strategies may be chosen to test for association in samples designed for linkage studies characterized by the presence of related affected individuals, from the random draw of a single case per family (considering either all families or a subset of linked families) compared to unrelated controls [1], to family-based association tests (FBAT) that use internal controls [2]. Recently corrections of classical case-control tests to allow the inclusion of related individuals have been proposed [3,4]. No comparison of these different strategies has been published yet. The Genetic Analysis Workshop 14 simulated problem provides an interesting data set to compare these methods. The analyses are being performed without knowledge of the answers. Three loci associated with the simulated Kofendrerd Personality Disorder (KPD) disorder were identified and then the power of different statistics considering various sampling strategies was studied. In this work, the single-nucleotide polymorphism map which covers the 10-chromosome genome with markers 3 cM apart was used, and the affection status for KPD provided by the physicians in each population was the trait of interest.

## Methods

In a first step we identified markers associated with KPD using sets of either 100 independent nuclear families with at least 2 affected offspring from the AI, KA, and DA populations or sets of 50 extended pedigrees with at least 4 affected members from the NY population. No independent control group was available at this stage. The quasi-likelihood score test for case-control association (CC-QLS) developed by Bourgain et al. [4] was used for case control association in samples with related individuals (see below for description) using the unaffected members of the families as controls. The test was performed separately in the 100 replicates of the four populations and for all the markers of the map. Loci with a nominal *p*-value ≤ 0.01 in at least six replicates per population, in three of the four populations were selected. Three loci met these arbitrary criteria: C03R0281, C05R0380, and C09R0765 (Table 1). A fourth locus, C10R0880, showed significant association in more than half of the replicates in two of the four populations. These results were confirmed in each population by randomly drawing one case per family in the 100 replicates, pooling them and comparing the frequencies of the 10,000 case sample (or 5,000 in NY) with the population frequencies provided by the organizers. C03R0281, C05R0380, and C09R0765 were highly associated with KPD (*p*-value ≤ 10^-8^) in the four populations, C10R0880, was highly associated with KPD (*p*-value ≤ 10^-8^) in the DA, KA, and NY population. Two additional loci (C01R0052 and C02R0097) were detected in AI. C01R0052 was also detected in DA and C02R0097 in KA.

The following steps focused on two populations with a roughly similar definition of the KPD phenotype (the proportion of KPD cases with each of the 12 characteristics associated with this disease are close in these two populations) but offering a different sampling scheme: AI (nuclear families) and NY (extended pedigrees). For this reason, only the loci detectable in single replicates of these two populations (C03R0281, C05R0380, and C09R0765) were considered.

In a second step, the powers of four different statistics to detect these three loci considering different sampling schemes were compared. Power was assessed as the observed proportion of replicates, in which the association could be detected using a nominal *p*-value of 0.01. Because the null distribution of the statistics compared have been shown to be chi-squared in various contexts of relatedness and on larger simulation sets [3-5], type I errors were assumed to be 1% for all statistics. Given the relatively low number of replicates available, the results should be considered very cautiously.

### FBAT

Proposed by Rabinowitz and Laird [2], it is a generalization of the transmission disequilibrium test (TDT) which allows, in particular, the analysis of sibships with multiple affected individuals or more general pedigrees. As pointed by Lake et al. [5], when analyzing pedigree data or multiple affected sibs in linked regions, a robust estimator of the variance of the score on which the statistic is based, should be used to perform valid tests. Like the TDT, FBAT can be expressed as a likelihood score test performed conditional on the founder's genotypes. It is thus robust to the presence of population stratification.

The three other tests considered in this paper are all unconditional tests for association that were used because no population stratification was present in the data.

### Corrected χ^2 ^test for case control association (CC-χ^2^_corr_)

Proposed by Bourgain et al. [4], it corresponds to a classical χ^2 ^test for allelic association corrected for the inter-individual correlations. Briefly, when considering non related samples, the classical χ^2 ^test can be expressed as a score test (χ^2 ^= S^2^/var(S), where S is a likelihood score). In the presence of inter-individual correlations, it is possible to compute the variance that appropriately accounts for these correlations. The corrected variance proposed here only depends on the known genealogical links between the cases and the controls of the sample.

### Quasi-likelihood score test for case control association (CC-QLS)

Also proposed by Bourgain et al. [4], it uses a similar approach but not only corrects the variance for the presence of related individuals but also the score (which in this case corresponds to a quasi-likelihood score). These authors have shown the CC-QLS test to be asymptotically the locally most powerful test of a class of statistics which includes the CC-χ^2^_corr_.

### Corrected trend test for association (corrIBD-trend)

Proposed by Slager and Schaid [3], it is similar to the CC-χ^2^_corr _but it is based on the Armitage trend test [6] for association and not on the allelic test. Further, whereas the correction of the variance is computed using the genealogical information for the CC-χ^2^_corr _and the CC-QLS, in the corr_IBD_-trend it is computed conditionally on the identity by descent (IBD) between all the individuals (cases and controls), estimated from the marker genotypes. In their paper, Slager and Schaid [3] proposed the method for the general situation of related cases and controls. However, their program can only handle unrelated controls, so the corr_IBD_-trend was used in this latter situation only. The program was extended to base the variance correction on multipoint IBD estimates, computed using GENEHUNTER [7], rather than single-point estimates.

### Sampling schemes

FBAT, CC-χ^2^_corr_, and CC-QLS were used on the initial family data considering all the affected individuals as cases. Non-transmitted parental alleles were used as controls in FBAT. All unaffected members of the nuclear families or extended pedigrees were the controls for both CC-χ^2^_corr _and CC-QLS.

CC-χ^2^_corr_, CC-QLS, and corr_IBD_-trend were used on samples consisting of all the affected individuals of the families and 200 unrelated controls. The unrelated controls were obtained after the ordering of the packs corresponding to the three loci studied and the pooling of four sets of controls.

CC-χ^2^_corr _and corr_IBD_-trend were used on samples consisting of a single case randomly drawn from each independent family and 200 unrelated controls. In this particular case, the CC-χ^2^_corr _is strictly equivalent to the CC-QLS and both correspond to the classical χ^2 ^for allelic association. The corr_IBD_-trend is strictly equivalent to the Armitage trend test for association.

The corr_IBD_-trend was applied to samples of cases selected on their IBD status. Indeed, because both CC-χ^2^_corr _and CC-QLS use an unconditional correction factor for the variance, these tests would be biased for samples selected upon the IBD status. Following Fingerlin et al. [1], samples made of cases from the families with a NPL_pairs _≥ 0 (NPL using S_pairs_) and 200 unrelated controls were used. The corr_IBD_-trend was used on either all the cases from these families or on a single case per family, randomly drawn among the affected sibs of each family. In this latter case, the corr_IBD_-trend corresponds to the Armitage trend test for association.

## Results

Table 2 presents the power with a nominal type I error of 1% for the three statistics available in the initial family data. The unconditional approaches of the CC-χ^2^_corr _and the CC-QLS are clearly more powerful than the conditional FBAT approach, although the gain in power varies with the locus (and thus the genetic model) and the sampling scheme (nuclear families versus extended pedigrees). In particular, for the most associated locus (C03R0281), the power of FBAT is significantly reduced in the extended pedigrees. This result likely reflects that the robust variance option proposed by Lake et al. [5] for FBAT may strongly affect power when considering extended pedigrees. Indeed, in the AI sample of independent nuclear families, FBAT and CC-χ^2^_corr _have nearly the same power. The interest in CC-χ^2^_corr _and the CC-QLS over FBAT is thus particularly meaningful while considering extended pedigrees. Further, as shown analytically [4], CC-QLS performs slightly better than CC-χ^2^_corr_. Table 3 presents the results when all the cases in the families and unrelated controls are considered. Surprisingly, the three statistics have equivalent power, for the two populations and the three loci. Indeed, because the variance in the corr_IBD_-trend is corrected using the genealogy and the marker information, this test is expected to be a more powerful test of association than a similar test in which the variance would be corrected using solely the genealogy. The corr_IBD_-trend test is an Armitage trend test and CC-χ^2^_corr _a chi2 test for allelic association, but this difference in nature of the test does not seem to be an explanation for the power results. Results presented in Table 5 outline that for the two populations and three loci, when considering unrelated individuals (a situation where the only difference between the two tests is their nature and not the information included in the variance correction), CC-χ^2^_corr _and corr_IBD_-trend have the same power. A possible explanation for the results in Table 3 is that, though less powerful to detect association, because it is unconditional, the corrected variance implemented in CC-χ^2^_corr _and CC-QLS additionally benefits from linkage when present, which is not the case of the conditional corrected variance. The mean IBD distribution among affected sibs from the AI population presented in Table 4 (one random pair per family) demonstrates that linkage is present for the three loci. The loss of power to detect association of the unconditional correction seems to be compensated by the use of linkage information. Whereas 200 controls are available in this sampling scheme versus >400 for AI and >600 for NY in the previous scheme, they are unrelated to each other and to the cases. Consequently, power is significantly increased. For locus C03R281 in AI, 200 unrelated controls provide a power 30% above the one obtained with >400 related controls. Were statistics allowing the sampling of multiple related cases not available, classical χ^2 ^test or trend test for association would have to be performed after selecting a single case per family. The power of these tests in both AI and NY when selecting a random case per family is shown in Table 5. The comparison with Table 3 shows that power is doubled in NY when using all cases instead of a random single case, and increased by a third in AI.

Finally, Table 6 displays the power in population AI of the sampling strategy where only cases from families with NPL_pairs _≥ 0 are selected, either a single random case per family or all of them. The comparison with Table 5 reveals that for loci C03R0281 and C05R0765, the power of this sampling scheme is equal or smaller than the power of the unselected scheme where all the families are included. This result holds whether a single random case or all of them are considered. However, the result is inverted for C09R0765, in the random single case. This difference could be explained by a different genetic model for this locus, which would change the best sampling strategy, but it could also just result from the low number of replicates available to compute the power, combined with a low power at this locus.

## Discussion

The method comparison was based on power rather than on a measure of efficiency that would correct for the number of subjects typed. Indeed, given the low power to detect risk factors for complex diseases, if typing all the available subjects rather than a subset creates a better chance to detect an association, investigators will certainly favor this strategy. The issue is thus, for a given sample of cases and controls, what is the best strategy to detect an association when present?

Although based on only 100 replicates, the power comparisons performed on the Genetic Analysis Workshop 14 data show for three different genetic models, that in the absence of unrelated controls and in non-stratified populations, unconditional tests for case-control association corrected for the presence of related individuals, such as the CC-QLS or the CC-χ^2^_corr_, are more powerful approaches to detect association using samples designed for linkage studies than "TDT-like" conditional approaches. Of course, both the CC-QLS and the CC-χ^2^_corr _only correct for relatedness. Therefore, they are not valid in the presence of population stratification and should only be considered when stratification is not suspected. Because they were initially developed for large inbred pedigrees in which IBD computations are not feasible, the corrections for the presence of related individuals implemented in both the CC-QLS or the CC-χ^2^_corr _are based solely on the genealogy. This analysis shows that even when IBD computations are feasible, incorporating this supplementary information in the correction does not systematically increase power. The possibility for tests based on an unconditional corrected variance to make use of linkage information when present, counterbalances the loss of power due to a coarser correction.

Finally, as expected, sampling strategies based on unrelated controls are the most powerful strategies, particularly when all the cases from all the families are included. The discussion of whether the power gained by typing all the cases from all the families rather than a single case from linked families is worth the cost increase remains open.

## Abbreviations

CC-χ^2^_corr_: Corrected χ^2 ^test for case control association

CC-QLS: Quasi-likelihood score test for case-control association

corrIBD-trend: Corrected trend test for association

FBAT: Family-based association test

IBD: Identity by descent

KPD: Kofendrerd Personality Disorder

TDT: Transmission disequilibrium test

## Figures and Tables

**Table 1:** Number of replicates with CC-QLS *p*-value < 0.01 in each population

	Population			
	
Marker	AI	DA	KA	NY
C03R0281	24	26	19	25
C05R0380	6	2	8	6
C09R0765	8	3	14	12
C10R0880	1	56	57	0

**Table 2:** Power of FBAT, CC-χ^2^_corr _and CC-QLS tests in the family data (internal controls)

	AI (269.23 cases/412.94 controls)			NY (273.73 cases/654.11 controls)		
		
Marker	FBAT	CC-χ^2^_corr_	CC-QLS	FBAT	CC-χ^2^_corr_	CC-QLS
C03R0281	17	18	24	8	25	25
C05R0380	1	2	6	6	5	6
C09R0765	3	3	8	3	7	12

**Table 3:** Power of corr_IBD_-Trend, CC-χ^2^_corr_, and CC-QLS tests with all the cases and unrelated controls

	AI (269.23 cases/200 controls)			NY (273.73 cases/200 controls)		
		
Marker	corr_IBD_-trend	CC-χ^2^_corr_	CC-QLS	corr_IBD_-trend	CC-χ^2^_corr_	CC-QLS
C03R0281	29	28	30	32	35	33
C05R0380	5	4	4	5	6	3
C09R0765	9	9	10	6	6	7

**Table 4:** IBD distribution in affected sib pairs of population AI

Marker	z_0_	z_1_	z_2_
C03R0281	0.175	0.476	0.349
C05R0380	0.179	0.482	0.339
C09R0765	0.194	0.465	0.341

(z_0_, z_1_, z_2_) are the proportions of sib pairs with IBD = 0, 1, or 2 estimated from the marker data

**Table 5:** Power of the CC-χ^2 ^and CC-Trend tests with a random case per family

	NY (50 cases/200 controls)		AI (100 cases/200 controls)	
		
Marker	CC-χ^2^_corr_	corr_IBD_-trend	CC-χ^2^_corr_	corr_IBD_-trend
C03R0281	17	18	23	25
C05R0380	4	3	4	3
C09R0765	2	3	8	8

**Table 6:** Power of the corr_IBD_-trend test with one or all cases from linked families

	AI			
	
	1 case		All cases	
		
Marker	No. cases	CC-trend	No. cases	corr_IBD_-trend
C03R0281	64,17	19	177,55	23
C05R0380	64,71	1	169,06	2
C09R0765	61,13	15	158,44	9

Cases are sampled from families with NPL_pairs _≥ 0
